# Enhancing *Ebony?* Common Associations With a *cis-*Regulatory Haplotype for *Drosophila melanogaster* Thoracic Pigmentation in a Japanese Population and Australian Populations

**DOI:** 10.3389/fphys.2018.00822

**Published:** 2018-07-10

**Authors:** Marina Telonis-Scott, Ary A. Hoffmann

**Affiliations:** ^1^School of Life and Environmental Sciences, Deakin University, Geelong, VIC, Australia; ^2^Pest and Environmental Adaptation Group, School of BioSciences, Bio21 Institute, The University of Melbourne, Melbourne, VIC, Australia

**Keywords:** *Drosophila*, pigmentation, *ebony*, *cis*-regulatory enhancer, evolution

## Abstract

The molecular underpinnings of pigmentation diversity in *Drosophila* have recently emerged as a model for understanding how the evolution of different *cis*-regulatory variants results in common adaptive phenotypes within species. We compared sequence variation in a 5′ regulatory region harboring a modular enhancer containing a ∼0.7-kb core element contributing to abdominal melanisation in African, and a ∼0.5-kb core element contributing to thoracic pigmentation in *D. melanogaster* from Japan, to tropical and temperate populations from eastern Australia previously shown to be divergent in thoracic pigmentation and *ebony* expression. The Australian populations exhibited strong association with the core enhancer polymorphism cluster in complete association with Dark and Light phenotypes from Iriomote, Japan. Moreover, the Iriomote Light and Dark core enhancer haplotypes are common to the Australian populations in the direction predicted by pigmentation phenotype. We also confirmed the Japanese patterns of linkage disequilibrium and association of the tropical inversion *In(3R)Payne* with the Light enhancer haplotype in the Australian tropical light population. A worldwide survey of the ∼0.5-kb *ebony* control region SNPs and haplotypes in a subset of the Drosophila Genome Nexus (DGN) populations suggest origins in the sub-Saharan ancestral region surrounding Zambia and subsequent invasion following colonization out of Africa. A previous study demonstrated complex within and between population genetic architecture for abdominal pigmentation which is also correlated with thoracic pigmentation in melanized DGN sub-Saharan populations; however, the ∼0.5-kb *ebony* control region was not associated and both haplotypes are common even in the most intensely pigmented *D. melanogaster* from high altitude Ethiopia. In the Australian populations, the strong phenotypic association with the enhancer SNPs and haplotypes that at least partly regulates *ebony* expression in the Iriomote population, our previous work demonstrating opposing clines for thoracic pigmentation and *ebony* expression, where the expression cline parallels the *In(3R)Payne* cline, and the concerted evolution of pigmentation intensity and *ebony* expression under rapid experimental evolution, all point to a common adaptive evolutionary pathway in distinct populations.

## Introduction

Pigmentation diversity is a widely studied model of adaptive phenotypic variation among different taxa. In *Drosophila*, pigmentation varies between species as well across geographical gradients in repeated clines, supporting spatially varying selection rather than demographic history in maintaining continuous variation in pigmentation intensity across populations (David et al., 1985; Hollocher et al., 2000; Brisson et al., 2005; Pool and Aquadro, 2007; Wittkopp et al., 2010; Telonis-Scott et al., 2011). Commonly proposed selective advantages for pigmentation variation include adaptive melanism, where darker pigments absorb greater solar radiation to improve thermoregulation (reviewed in True, 2003; Clusella Trullas et al., 2007; Rajpurohit et al., 2008), although flies are likely too small to benefit in heat budgeting. Rather differences in melanism in flies may confer greater tolerance to desiccation and UV protection (Parkash et al., 2008; Matute and Harris, 2013; Bastide et al., 2014).

In *Drosophila melanogaster*, the striking morphological diversification in body coloration has been attributed to a number of key genes in the melanin synthesis pathway including *yellow*, *tan*, and *ebony* (Wittkopp et al., 2003; Massey and Wittkopp, 2016). Fine dissection of the transcriptional control of *ebony* has revealed complex evolution of intraspecific *cis*-regulatory variation (Rebeiz et al., 2009; Takahashi and Takano-Shimizu, 2011; Miyagi et al., 2015). Rebeiz et al. (2009) demonstrated that regulatory divergence at *ebony* in Ugandan *D. melanogaster* is restricted to a 5′ regulatory region harboring a 2.4-kb modular enhancer containing the 0.7-kb core abdominal element, *e*_abdominalCRE, where at least five newly arisen functional mutations contribute to an allele of large effect on abdominal melanism. Takahashi and Takano-Shimizu (2011) investigated the effect of a 13-kb region including the *e*_abdominalCRE on thoracic pigmentation in dark and light inbred strains from a color variable population from southern tropical Japan. At least part of the expression divergence was likely due to *cis*-regulatory mutations in this region but not within the *e*_abdominalCRE (Rebeiz et al., 2009), where 11 out of 19 sites in complete association with the dark and light phenotypes clustered within ∼0.5 kb of the core abdominal enhancer in strong linkage disequilibrium (LD; Takahashi and Takano-Shimizu, 2011). Moreover, the Light-type enhancer haplotype was strongly linked to the tropical inversion *In(3R)Payne*, where seven derived nucleotide sites clustered within ∼0.5 kb were also in strong LD. Miyagi et al. (2015) assessed the effects of *cis-*regulatory variants of four genes in the melanin biosynthesis pathway using allele specific analysis of a subset of the *D. melanogaster* Genetic Reference Panel (DGRP). Miyagi et al. (2015) examined ∼10 kb of the *ebony cis*-regulatory region (Rebeiz et al., 2009; Takahashi and Takano-Shimizu, 2011) and found *ebony* in strong association with the thoracic trident phenotype in multiple variants outside but not within the core enhancer.

These studies emphasize the lack of overlap between causal or associated polymorphisms and pigmentation intensity at either the *ebony* core enhancer or in sequences flanking the control region. This points to multiple *cis*-regulatory variations on the genetic path to common phenotypes. Perhaps not surprisingly, more relaxed constraints operate on evolving regulatory sequences including individual modular enhancers (Rebeiz et al., 2009), although Miyagi et al. (2015) suggest that intraspecific differences controlling pigmentation are more complex than those causal changes restricted to modular enhancers controlling differences between species.

Previously, in east coast Australian *D. melanogaster*, we characterized temperate–tropical latitudinal clines of thoracic pigmentation intensity and *ebony* expression at 25°C (Telonis-Scott et al., 2011), which also parallels the cline of *In(3R)Payne* (reviewed in Hoffmann and Weeks, 2007). *Ebony* expression accounted for ∼70% of the phenotypic variation across the 18 east coast populations sampled, supporting *ebony* at least partially contributing to pigmentation diversity in Australian *D. melanogaster* (Telonis-Scott et al., 2011). Here, we examined sequence diversity associated with pigmentation intensity and *ebony* transcript expression in the strongly supported ∼0.5 kb *ebony* control region in a tropical and temperate population representing cline-end populations diverged for both phenotype and gene expression. The Australian outbred cline-end populations showed strong SNP associations with Dark and Light Iriomote phenotypes; the Japanese Dark polymorphisms always segregated at very high frequency in the Australian temperate (dark) population, and *vice versa* for the Japanese Light polymorphisms and the Australian tropical (light) population. We also demonstrate association of the divergent haplotypes in the direction predicted by the Japanese phenotypes as well as inter-population variation, and found the Light haplotype in association with *In(3R)Payne* in the light tropical Australian population, supporting the maintenance of the divergent haplotypes by climatic selection in the Australian populations. A cross-continent survey revealed the presence of the SNPs and haplotypes in both cosmopolitan and sub-Saharan African populations, suggesting origins in the *D. melanogaster* ancestral region around Zambia, and a lack of clear association with the varied African phenotypes.

## Materials and Methods

### Australian East Coast *D. melanogaster* Populations

The cline end populations used in this study [INS: Innisfail QLD (17.52°S) and TAS: Sorrell TAS (43.15°S)] were sampled from a larger collection of 18 locations along the Australian east coast in April–June 2008 (see Sgrò et al., 2010).

### Thoracic Trident Pigmentation Scoring and Ebony Expression Assays

Fly rearing, trident pigmentation phenotyping, and *ebony* gene expression assays performed at 25°C are described in Telonis-Scott et al. (2011). Thoracic trident pigmentation was scored by visual examination using four phenotypic classes described by David et al. (1985), and *ebony* expression was quantified from the thoraxes of female imagoes by relative quantification using real-time PCR (Telonis-Scott et al., 2011).

### Ebony Core Epidermis Control Region Sequencing

The east coast Australian cline ends exhibited the darkest (TAS, temperate population) and lightest (INS, tropical populations) trident phenotypes corresponding with low and high *ebony* transcript abundance, respectively (average pigmentation score: TAS = 1.10, INS = 0.02, relative ebony expression: TAS = 0.19, INS = 0.45; Telonis-Scott et al., 2011). DNA was extracted from 25 individuals from each population using the High Pure PCR Template Preparation Kit (Roche) following the manufacturer’s instructions. 545 bp of the ebony ∼0.5 kb core epidermis control region was amplified from 25 individuals from each population using the primer sequences: 5′–3′ TCGGTTCTCAGGTGCTTTTT, and 3′–5′ TCACAGGGACTTTTGGGAAA. Agarose gel electrophoresis (3.5%) was used to screen flies for indels, and heterozygotes with two prominent bands excised separately on a 5% acrylamide gel and used as the PCR templates for sequencing. Several bands proved difficult to amplify and were amplified again using ^33^P labeled primer. Both strands were sequenced using Sanger sequencing (Macrogen, Korea). Sequences were aligned using ClustalW (Larkin et al., 2007) using the consensus Iriomote Light and Dark sequences (Takahashi and Takano-Shimizu, 2011) as the reference sequences. We obtained largely complete 545 bp core enhancer sequences (positions 761–1305 from Takahashi and Takano-Shimizu, 2011, including 10 of the 11 sites in complete association with the Japanese Light and Dark phenotypes clustered around the core enhancer) for 19 INS and 15 TAS individuals, respectively. Fisher’s exact tests were used to compare allele frequencies at 34 polymorphic sites, and *P* values were corrected for multiple tests using the false discovery rate (FDR) method (Benjamini and Hochberg, 1995). Pairwise LD was measured and the heatmap generated with LDheatmap (Shin et al., 2006) implemented in R.

### *In(3R)Payne* Screening and Sequencing

The populations were screened for *In(3R)Payne* following Anderson et al. (2005) and Rane et al. (2015). Briefly, a 570-bp region close to the proximal break point was amplified using the following primers: In(3R)P-SNP_outer_1; 5′–3′ TTTGCCGCAAATTATTGTGAG and In(3R)P-SNP_outer_2; 3′–5′ ATCGCGTGCAGGTTGGC (Anderson et al., 2005). The PCR amplicons were sequenced and individuals scored using the diagnostic SNP assay (G = inverted, A = standard arrangement; Rane et al., 2015). All 25 individuals from each population were screened and unambiguous arrangements were obtained for 20 TAS and 11 INS samples. Inversion frequencies were compared using Fisher’s exact test.

### Survey of Worldwide *D. melanogaster* Population SNP and Haplotype Distribution

To examine the Iriomote divergent haplotypes in a population genetics context in comparison to the Australian sequences, the core enhancer SNP, haplotype (including indels), and *In(3R)P* distributions were examined in 19 populations spanning four continents: Oceania (1), America (2), Europe (2), and Africa (14), using a population subset of genome sequences from the Drosophila Genome Nexus (DGN) *Version 1.1* (Lack et al., 2015, 2016). However, there are several caveats limiting population genetic analyses for this region; therefore, we utilize the data to survey variation in this region while tentative inferences are based on previously published data utilizing entire genomes. First, sequencing depth varies substantially among genomes impacting site calling in difficult regions including indels, and bias exists in read mapping due to higher relatedness of some genomes to the reference strain. More importantly, the DGN pipeline masked sites within 3 bp of indels relative to the reference sequence (Flybase release 5 of the *D. melanogaster* genome) and indels were clipped from the alignments. Sites that failed to meet alignment base quality thresholds were filtered to *N*, and genomic regions with residual heterozygosity (or pseudo-heterozygosity from haploid genomes) were entirely masked.

The sequences of the core *ebony* enhancer (coordinates 3R:17066613…17067587) were extracted using PopFly (Hervas et al., 2017). Sequences from each population were aligned separately and haplotypes scored as above. Due to the DGN filtering pipeline, several individuals from each population were missing data for this region. We included populations with at least four individuals and low regions of missing data.

Separate VCF variant files summarizing the indels were obtained from the DGN and converted to .csv files to examine indel status. There are also several caveats with the indel mapping and the authors caution that incongruities between round one and two indels calls were not resolved and the files are intended only to check if indels exist in the region of interest.

## Results

### Phenotypic and Polymorphism Overlap Between Australian and Japanese Populations at the Core Enhancer

We screened the ebony 5′ ∼0.5 kb core enhancer region (Takahashi and Takano-Shimizu, 2011) for polymorphisms associated with the divergent pigmentation phenotypes between two natural cline-end populations from temperate (TAS) and tropical (INS) East Australian *D. melanogaster*. Sequence analysis of 19 and 15 individuals (INS and TAS, respectively) revealed 34 polymorphic sites (including 3 indels) spanning positions 804–1299 (note, we obtained sufficient sequence for positions 804–1299 but not 1320, from Takahashi and Takano-Shimizu, 2011). Polymorphisms at 9/10 nucleotide sites and one indel (positions 999–1028) matched the core clustered enhancer sites in complete association with the Dark and Light phenotypes among inbred strains from Iriomote, Japan (Takahashi and Takano-Shimizu, 2011; **Table 1**). The TAS and INS populations differed significantly in allele frequencies in the direction expected based on the Dark and Light phenotype alleles (Fisher’s exact test, **Table 1**). The Dark alleles associated at high frequency in the dark TAS population (**Table 1**), while the Light alleles including the 30-bp insertion associated at high frequency in the light INS population (**Table 1**). Several other polymorphisms present in the control region but not in complete association in the Japanese population were also present in the Australian populations, again in the direction expected by Light and Dark phenotypes. These were position, pop (allele frequency): 1072, TAS, T (0.54) versus INS, A (0.93), FDR 0.001; 1149, TAS,T (0.83), versus INS,T(1), FDR < 0.05; 1158, TAS,C (0.67) versus INS,C (0.08), FDR < 0.05; 1290: TAS,C (0.86) versus INS,C (1), FDR < 0.06.

**Table 1:** Overlap of segregating Australian, and fixed Japanese polymorphisms associated with the dark and light phenotypes from Iriomote Japan clustered around the ∼0.5-kb core enhancer in the ∼0.9-kb epidermis control region [e_ERC0.9, positions 804–1299 from Takahashi and Takano-Shimizu (2011)].

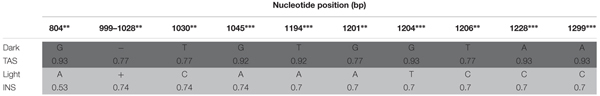

*The consensus haplotypes of the Iriomote Dark (dark gray shading) and Light (light gray shading) strains are shown, and the corresponding major allele frequencies from the Australian dark temperate population (TAS, dark gray shading: *n* = 15) and light tropical population (INS, light gray shading: *n* = 19). Differences in allele frequencies between TAS and INS were tested using Fisher’s exact test with FDR correction for multiple tests. ^∗∗∗^FDR < 0.0001; ^∗∗^FDR < 0.01.*

### Divergent Enhancer Haplotypes

The Light and Dark enhancer haplotypes (characterized by 11 fixed differences within the core enhancer; Takahashi and Takano-Shimizu, 2011) were present in both Australian tropical/temperate populations. In the dark TAS population, the Dark haplotype predominated (nine homozygous individuals), and the Light haplotype was rare (one homozygous individual; **Supplementary Table S1**). The remaining five individuals retained half of the Dark combination (positions 804, 1194, 1204, 1228, and 1299, **Supplementary Table S1**) and were Dark/Light heterozygotes for the other core enhancer sites (positions 999–1028, 1030, 1045, 1201, and 1206, **Supplementary Table S1**). Flies from the light population INS were more variable at the enhancer, with five individuals homozygous for the Light enhancer haplotype, five individuals homozygous for the Light haplotype barring a Dark G substitution at position 804, one individual homozygous for the Dark haplotype, and the remaining eight individuals being Dark/Light heterozygotes (**Supplementary Table S1**). Flies from either population harboring the Dark or Light haplotype (including the Light haplotypes with the G at position 804) were not polymorphic at any sites outside of the core cluster, with the exception of one light individual with an indel (positions 1154–1158, **Supplementary Table S1**).

### Australian Polymorphisms Outside the Core Cluster

The Australian polymorphisms comprise an additional 22 nucleotide sites and two population specific indels (**Tables 2**, **3**). Notably, the Australian polymorphisms segregated with the heterozygotes, with the exception of three Light haplotype INS flies (A/T at site 934, **Table 2**; **Supplementary Table S1**). Most of the variation (14 sites) was found in the five dark TAS heterozygotes, with only two polymorphisms in light INS flies (**Table 2**). The TAS specific indel (positions 1137–1158, **Table 3**) was also found in the U76^(3)^ strain whose sequences were shown to drive reporter gene expression in the abdominal epidermis (Rebeiz et al., 2009). No other sites overlapped with the core element polymorphisms from Rebeiz et al. (2009). The second smaller indel specific to INS was occurred within the above indel (positions 1154–1158, **Table 3**).

**Table 2:** Australian polymorphisms in 0.545 kb of the 0.9-kb epidermis control region [e_ERC0.9, positions 804–1299 from Takahashi and Takano-Shimizu (2011)].

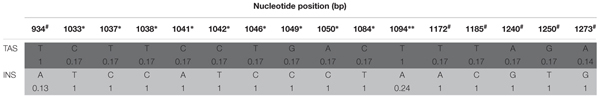

*Polymorphisms are shown with the corresponding allele frequencies from the Australian dark temperate population (TAS, dark gray shading: *n* = 15) and light tropical population (INS, light gray shading: *n* = 19). Differences in allele frequencies between TAS and INS were tested using Fisher’s exact test with FDR correction for multiple tests. ^∗∗^FDR < 0.01; ^∗^FDR < 0.05; **^#^**FDR = 0.06.*

**Table 3:** Indels in 0.545 kb of the 0.9-kb epidermis control region [e_ERC0.9, positions 804–1299 from Takahashi and Takano-Shimizu (2011)].

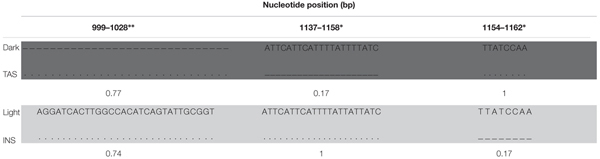

*The consensus indels of the Iriomote Dark (dark gray shading) and Light (light gray shading) strains are shown, and the corresponding allele frequencies of the Australian dark temperate population (TAS, dark gray shading: *n* = 15) and light tropical population (INS, light gray shading: *n* = 19). “-” indicates a base deletion and “.” indicates an identical base in the Australian populations relative to the Japanese populations. Differences in allele frequencies between TAS and INS were tested using Fisher’s exact test with FDR correction for multiple tests. ^∗∗^FDR < 0.01; ^∗^FDR < 0.05.*

### Patterns of Linkage Disequilibrium (LD)

**FIGURE 1:**
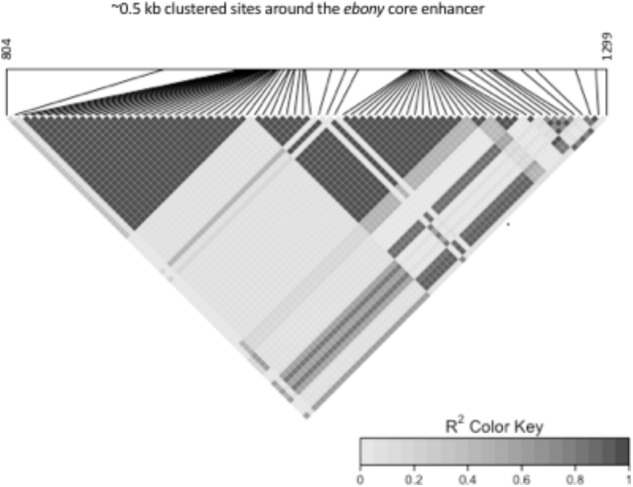
Pairwise LD *r*^2^ heatmap of 82 sites (sites 804 and 1299 are shown as first and last nucleotide positions) in 545 bp of the *ebony* core enhancer.

We observed a high degree of LD in the ∼0.5 kb core enhancer region: all Japanese Light and Dark alleles in complete association with the Dark and Light phenotype (Takahashi and Takano-Shimizu, 2011) exhibited LD in the Australian populations. The 999–1028 bp deletion was in LD with all sites varying in strength of association. The LD block in highest association included sites, 1201, 1204, 1206, 1228, and 1229 (**Figure 1**). The second LD block predominantly comprised the Australian specific alleles, with several nucleotide sites and the two indels exhibiting complete LD (**Figure 1**).

### *In(3R)Payne* Is Associated With the Light Haplotype

It is well established that the inversion *In(3R)Payne* clines across the Australian east coast, with frequencies highest at tropical low latitudes, decreasing to almost absent in high latitude temperate populations (Hoffmann and Weeks, 2007). We screened all 25 individuals each from the dark TAS and light INS populations, and for individuals where the SNP assay was unambiguous, confirmed that the inversion is at highest frequency in tropical INS (*n* = 11), while the standard arrangement is predominant in temperate TAS (*n* = 20; 0.82 and 0.88, respectively, Fisher’s exact test *P* < 0001).

We next calculated inversion frequencies from only those individuals used for the sequence analysis and found a higher frequency of the inversion among INS individuals (*n* = 8) and higher frequency of the standard arrangement in TAS (*n* = 13; 0.94 and 0.92, respectively, Fisher’s exact test *P* < 0001). The inversion was present in the 10 individuals from INS with the Light, or predominantly Light haplotype and heterozygous in one Dark/Light haplotype heterozygote. In TAS, it was only present in the one individual with the Light enhancer haplotype (**Supplementary Table S1**).

### Cross-Continent Survey of Core Enhancer Cluster SNPs and Haplotypes

We utilized the DGN (Lack et al., 2015, 2016) to examine within and between population sequences at the core *ebony* enhancer across four continents with the majority of populations from four sub-Saharan African subclans increasing in latitude towards the south (13 populations) and 6 cosmopolitan populations ranging from mid-high latitude (**Table 4**). The DGN alignment pipeline imposed several limitations on complete sequence capture in this region (see “Materials and Methods”); however, the polymorphisms and divergent haplotypes associated with the dark and light trident phenotypes and *ebony* expression in Japanese and Australian populations could be largely reconstructed and are found to occur worldwide. Note that the Light haplotype is *assumed* to be “Light” despite missing data at site 1030 flanking the Light insertion (sites 999–1028). Missing data at other core sites pervade the sequence alignments, thereby resulting in low region coverage and sample size; therefore, we summarize the data rather than attempt population genetic analyses likely confounded by sample bias. SNPs and haplotypes at the core enhancer are provided in **Supplementary Table S2**. All SNPs were observed in varying combinations in all populations, and both the Dark and Light haplotypes segregated in all populations except Cameroon, Uganda, and Winters, United States.

**Table 4:** Survey of previously published (DGN project) *D. melanogaster ebony* core enhancer sequence haplotypes, *In(3R)P* inversion status (denoted as INV) and the deletion (sites ∼1137–1158) in the U76^(3)^ strain whose sequences were shown to drive reporter gene expression in the abdominal epidermis (Rebeiz et al., 2009).

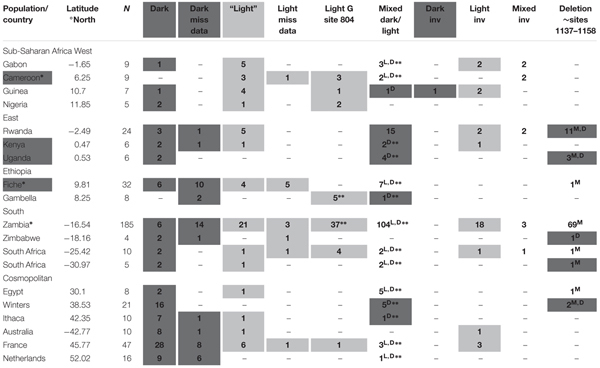

*The numbers of individual flies of each sequence category are shown in bold. “DARK” indicates the Iriomote consensus haplotype from Takahashi and Takano-Shimizu (2011) and the present study. “LIGHT” indicates the divergent Iriomote consensus haplotype including the insertion (sites 999–1028). The DGN alignment protocol resulted in several filtering steps producing missing data for most populations (see “Materials and Methods”). Notably, site 1030 is clipped 3 bp from the 999–1028 insertion, therefore the haplotype is called as “LIGHT” with caution. The numbers of Light haplotypes with the DARK G substitution at site 804 observed in the current study are included, and Mixed DARK/LIGHT refers to combined haplotypes with varying degrees of mixture. ^∗^Following Bastide et al. (2014), the sub-Saharan populations are grouped into four subclans according their genetic structure. The dark/light shading indicates melanized populations (dark) and the light ancestral population, Zambia (Bastide et al., 2016). ^∗∗^Missing data, D, DARK biased SNPs; L, light biased SNPs; M, mixed SNPs.*

In the sub-Saharan populations, the Light haplotype was more frequent in the west subclan including equatorial Cameroon and Guinea (**Table 4**). The Light haplotype with the Dark G substitution at position 804 observed in the Australian tropical Light population INS was also present in Cameroon, Guinea, and Nigeria, and combinations of the Dark/Light haplotypes were present in Gabon, Cameroon, and Guinea (**Table 4**). The Light haplotype was less frequent in the East equatorial population Kenya and absent in Uganda, and the mixed individuals were biased to Dark SNPs (**Table 4**). Most Rwandan individuals (15/24) harbored mixed SNPs to varying degrees, with similar numbers of Light and Dark haplotypes (**Table 4**).

We examined two populations from Ethiopia, including the highland population Fiche where flies exhibit a unique, intensely melanized trident phenotype undocumented in any African or cosmopolitan *D. melanogaster* or *D. simulans* population (Bastide et al., 2014). Both haplotypes were present in Gambella, Ethiopia individuals, although with more missing data and slightly biased to dark SNPs and mixed individuals (**Table 4**). By contrast, the Light haplotype with the Dark G substitution at position 804 was most present in the Fiche population (**Table 4**).

Southern Africa is likely the ancestral region of *D. melanogaster*, where Zambia is the most highly sampled and deeply sequenced DGN population given the highest levels of genetic diversity with minimal levels of admixture from cosmopolitan populations (Pool et al., 2012). As such, all haplotypes were present with a bias towards the Light and Light G substitution, although almost half of the flies exhibited diverse mixtures of the SNPs (104/185, **Table 4**). The small samples from Zimbabwe and South Africa (4 and 5, respectively) show both Light and Dark haplotypes and mixed individuals (**Table 4**). The larger South African population (*n* = 10) is Light biased with most individuals Light G 804 substitution (**Table 4**).

Barring Egypt where more mixed SNP individuals were observed, the cosmopolitan populations tended towards more Dark haplotypes, although with at least one Light haplotype and individuals with mixtures of the SNPs (**Table 4**). The Australian population sampled from the same site in Tasmania (Sorrell) as in the current study shows similar results, and the population from Winters, United States, had no Light haplotype and individuals with mixed SNPs were Dark biased. While the majority of individuals from France were Dark, Light haplotypes and one individual with the Light G 804 substitution were observed, perhaps due to the larger sample size (*n* = 47, **Table 4**).

### Cross-Continent *In(3R)Payne* and Indel Survey

We obtained the *In(3R)Payne* arrangements for almost every individual from each population from the DGN resources. While *In(3R)Payne* is most present with the Light haplotype, there was no complete association with the Light haplotypes and numbers were low even in Zambia where the most Light/partial Light haplotypes were observed (**Table 4**). Moreover, Nigeria, Uganda, Ethiopia, Zimbabwe, South Africa, Egypt, and Ithaca (NY, United States) all harbor Light haplotypes but no inversion (**Table 4**). *In(3R)Payne* is present in only one dark individual from Guinea west Africa (**Table 4**).

The U76^(3)^ strain deletion (positions 1137–1158, **Table 3**) observed at low frequency in the dark Australian population (TAS) and in truncated form in the light population (INS) is present in all Southern African populations at highest frequency in Zambia (69/197, **Table 4**) and is also present in around half the individuals from Kenya and Uganda, East Africa (**Table 4**). Of the cosmopolitan populations, only one and two individuals from Egypt and Winters California, respectively, harbor this deletion (**Table 4**). Notably, the deletion segregated in individuals with combinations of the enhancer SNPs rather than with Dark/Light haplotypes, and we found the deletion only in the TAS Dark heterozygotes (**Table 3**).

## Discussion

We examined core *ebony* enhancer polymorphisms, associated with thoracic pigmentation intensity in Dark and Light strains in a tropical Japanese population (Iriomote), in two Australian populations sampled from the ends of the opposing latitudinal cline for thoracic pigmentation and *ebony* expression (Telonis-Scott et al., 2011). The two Australian populations represent the geographic and phenotypic extremes of a cline, where temperate TAS flies are darkly pigmented and express low levels of *ebony* transcripts, and the tropical INS population flies are lightly pigmented and highly express *ebony* (Telonis-Scott et al., 2011). We found that the Iriomote polymorphisms at the 10 sites surveyed in our focal region of the ∼0.5 kb core enhancer cluster (Takahashi and Takano-Shimizu, 2011) are in strong association in the Australian populations in the direction predicted by the Iriomote phenotypes.

Moreover, the divergent haplotypes in complete association with the Dark and Light phenotypes from Iriomote were similarly associated in the Australian populations. The Dark Iriomote haplotype predominated in TAS individuals in complete association with the core enhancer cluster, while the Light Iriomote haplotype predominated in INS, either in individuals in complete association or as a partial haplotype (Dark G substitution, position 804). Interestingly site 804 was shown by precise mutagenesis to have no effect on abdominal *ebony* expression in African populations (site 186; Rebeiz et al., 2009). The homozygous Dark and Light haplotypes were monomorphic in the core enhancer region (except for one INS individual).

Our wild sampled populations were naturally variable for both melanization and *ebony* expression; however, low frequency variants or intermediate phenotypes were likely swamped by the majority of individuals sampled as evidenced by dark and light trait means (TAS and INS, respectively; Telonis-Scott et al., 2011). This variation was also present at the sequence level: both Dark/Light haplotypes are maintained in the Australian populations at low frequency (i.e. one individual of the opposite haplotype). We also observed Dark/Light heterozygote core enhancer cluster haplotypes segregating in the light population, INS, while several TAS individuals harbored a combination of homozygous Dark sites and heterozygous Dark/Light sites. Takahashi and Takano-Shimizu (2011) did not observe *cis*-regulatory effects on expression in heterozygotes that shared identical putative *trans*-regulatory factors. Whether at least some of the Australian expression and phenotypic divergence is due to variation in the *cis*-regulatory region, perhaps contributing to variation in heterozygous individuals has not been determined, and factors acting in *trans* are unknown in the Australian populations.

*Ebony* (93C) is located within *In(3R)Payne* (89C-96A), and therefore, it is not surprising that the inversion is also associated with the Light haplotype in the Australian tropical light population given its prevalence in tropical populations here and in other studies (Hoffmann and Weeks, 2007). The parallel inversion and *ebony* expression clines and the consistency observed here with Takahashi and Takano-Shimizu (2011) suggest an association between *ebony* expression level and *In(3R)Payne* frequency along the Australian cline. The mixture of Dark and Dark/Light heterozygous sites and greater surrounding nucleotide variation for five dark TAS flies compared to the INS Light/Dark heterozygotes could be due to some recombination between the higher frequency standard and rare inverted arrangements (Kennington et al., 2006); conversely, the INS heterozygous haplotypes may remain in complete linkage due to the high frequency of *In(3R)Payne*.

The common haplotype associations could theoretically arise from convergent evolution following invasion of *D. melanogaster* into both countries, or else invasion of both countries by a common haplotype. The latter scenario is more likely given the expansion of *D. melanogaster* out of equatorial Africa from the possible southern ancestral range around Zambia, where the greatest genetic diversity and minimal level of non-African admixture is observed (Pool et al., 2012; Lack et al., 2015, 2016), and where the Light/Dark SNPs, divergent core enhancer haplotypes, and *In(3R)Payne* are also most common. We found the haplotypes throughout sub-Saharan Africa and among the cosmopolitan populations, suggesting the migration of these haplotypes out of Africa. *D. melanogaster* has been present in Australia for over 100 years (Hoffmann and Weeks, 2007) following possible colonization from the north (Kennington et al., 2003). The species would have likely been present in Japan for an even longer time due to ongoing trade. Colonization outside of Africa has required adaptation to a range ecological factors that vary with latitude particularly in the derived high latitude populations such as Tasmania (Adrion et al., 2015). Pigmentation variation is such an example; in contrast to positive cosmopolitan pigmentation latitudinal clines, both thoracic and abdominal pigmentation are negatively correlated with latitude in sub-Saharan Africa with peak pigmentation in low latitude montane Ethiopia (Bastide et al., 2014).

To our knowledge there is no evidence to support an association between the divergent haplotypes and trident pigmentation in sub-Saharan Africa. Moreover the distribution of the haplotypes is unclear given the Light haplotype is common in low latitude sub-Saharan Africa including the intensely melanized highland Ethiopian population Fiche, but segregating in lowland Zambia where flies are light and are presumably not under directional selection for pigmentation (Bastide et al., 2014). Moreover, *In(3R)Payne* tends to segregate at lower frequency than the Light haplotypes among the populations, and is not associated with Light haplotypes from Nigeria, Ethiopia, Cameroon, Zimbabwe, one South African population, and Egypt. The evolution of melanism in response to diverse habitats in sub-Saharan Africa appears complex, and abdominal and thoracic pigmentation is positively associated in the African DGN populations (Bastide et al., 2014), suggesting at least some shared genetic basis, but this is likely to vary depending on adaptation processes specific to different populations. A bulk segregant analysis of the genetic basis of abdominal pigmentation within and between three melanic flies in DGN populations from Ethiopia, Cameroon, and Uganda crossed to the light ancestral strain from Zambia identified QTL harboring key pigmentation genes, but this varied both within and between populations, demonstrating a complex pigmentation genetic architecture (Bastide et al., 2016). Individual SNPs at *ebony* were differentiated in one and two crosses from Uganda and Ethiopia, respectively, with the most differentiated Ethiopian SNP falling in the first intron (Bastide et al., 2016), known to coregulate *ebony* expression in concert with *e*_abdominalCRE (Rebeiz et al., 2009).

The evolution of different core enhancer sites of *ebony* expression has been experimentally proven to affect different body regions (Pool and Aquadro, 2007; Rebeiz et al., 2009; Takahashi and Takano-Shimizu, 2011). On the other hand, Miyagi et al. (2015) found no core enhancer sites associated with the DGRP allele specific ratio and thoracic pigmentation but rather many putative *cis-regulatory* alleles outside the core enhancer with potential interacting elements dispersed across the ∼10-kb *cis*-regulatory region. Rebeiz et al. (2009) also identified other functional mutations outside the core enhancer for Ugandan abdominal pigmentation. The DGRP may vary from other cosmopolitan populations given North American *D. melanogaster* result from admixture between Africa and Europe, where the DGRP ancestry is estimated upwards of 20% African (Pool, 2015). It is also possible that other loci acting in *trans* may be located in *In(3R)Payne* impacting the Japanese phenotypes (Takahashi and Takano-Shimizu, 2011).

Nonetheless, several lines of evidence indicate that *ebony* is likely a common target of selection among populations of *D. melanogaster* from different geographies. Despite gene flow between east coast Australian populations (Kennington et al., 2003), the predominance of divergent haplotypes and patterns of linkage in both the inverted and standard arrangements suggest the maintenance of these haplotypes at a core *cis*-regulatory enhancer by spatially varying selection due to different environmental pressures. Furthermore, the allele frequency differences in the core enhancer between INS and TAS are consistent with previous work demonstrating a rapid response to forward and reverse selection for light and dark phenotypes and corresponding *ebony* expression in tropical INS, but not in temperate TAS, suggestive of past directional selection for darker pigmentation (Telonis-Scott et al., 2011). The overlap with Iriomote haplotypes previously shown to at least partly regulate *ebony* expression in the thoracic epidermis does not confirm the mode of action of *ebony* regulation in the Australian populations but is at least a strong example of intraspecific repeatability at the phenotypic, transcriptional and nucleotide level, and provides a future line of research to understand the intra-species evolution of a complex and variable *cis*-regulatory region.

## Author Contributions

MT-S and AH designed the experiments. MT-S performed the data collection and analysis. MT-S wrote the manuscript and AH contributed to the manuscript.

## Conflict of Interest Statement

The authors declare that the research was conducted in the absence of any commercial or financial relationships that could be construed as a potential conflict of interest.

## Supplementary Material

TABLE S1 Haplotype status of the east coast Australian populations in ∼0.5 kb core ebony abdominal enhancer (positions 804–1299 from Takahashi and Takano-Shimizu (2011)). INS, light tropical Innisfail population; TAS, dark temperate Tasmamania population. The genotypes of the strains used for the ebony enhancer activity assay TW1(G23) (dark strain) and Mel6(G59) (light strain) are shown for reference. The presence or absence of the inversion *In(3R)Payne* are shown for those individuals were the SNP assay yeilded data.

TABLE S2 This file contains the sequence alignments, polymorphic sites and Dark/Light SNPs and haplotypes for the 545 bp ebony core enhancer sequence in the 19 populations examined from the Drosophila Genome Nexus (DGN). Three sheets are provided for each population containing the alignments, haplotypes polymorphic sites. The sequences of the core ebony enhancer including the reference sequence were extracted using PopFly (coordinates 3R 17066613…17067587 FlyBase release 5 of the *D. melanogaster* genome and aligned using the PopFly reference sequence and sequences used for the ebony enhancer assay Iriomote TW1(G23) = DARK and Mel6(G59) = LIGHT (Takahashi and Takano-Shimizu, 2011). The sheets ending in 0.1 (_alignment) show the sequence alignments using the DGN reference and DARK and LIGHT sequences as the reference sequences. The sheets ending in 0.2 (L_D_haplotypes) also show the Inv *(3R)P* inversion status for each individual in the first column. The sheets ending in 0.3 show the SNPs in the core enhancer region. Note that the insertions and the surrounding 3 bp are clipped from the files and indels are coded as N and were scored from separate files as described in the methods.
